# An Assessment of Lexical, Network, and Content-Based Features for Detecting Malicious URLs Using Machine Learning and Deep Learning Models

**DOI:** 10.1155/2022/3241216

**Published:** 2022-08-25

**Authors:** Malak Aljabri, Fahd Alhaidari, Rami Mustafa A. Mohammad, Samiha Mirza, Dina H. Alhamed, Hanan S. Altamimi, Sara Mhd. Bachar Chrouf

**Affiliations:** ^1^Department of Computer Science, College of Computer and Information Systems, Umm Al-Qura University, Makkah 21955, Saudi Arabia; ^2^SAUDI ARAMCO Cybersecurity Chair, Department of Computer Science, College of Computer Science and Information Technology, Imam Abdulrahman Bin Faisal University, P.O. Box 1982, Dammam 31441, Saudi Arabia; ^3^Department of Networks and Communications, College of Computer Science and Information Technology, Imam Abdulrahman Bin Faisal University, P.O. Box 1982, Dammam 31441, Saudi Arabia; ^4^Department of Computer Information Systems, College of Computer Science and Information Technology, Imam Abdulrahman Bin Faisal University, P.O. Box 1982, Dammam 31441, Saudi Arabia; ^5^SAUDI ARAMCO Cybersecurity Chair, Department of Computer Engineering, College of Computer Science and Information Technology, Imam Abdulrahman Bin Faisal University, P.O. Box 1982, Dammam 31441, Saudi Arabia

## Abstract

The World Wide Web services are essential in our daily lives and are available to communities through Uniform Resource Locator (URL). Attackers utilize such means of communication and create malicious URLs to conduct fraudulent activities and deceive others by creating deceptive and misleading websites and domains. Such threats open the doors for many critical attacks such as spams, spyware, phishing, and malware. Therefore, detecting malicious URL is crucially important to prevent the occurrence of many cybercriminal activities. In this study, we examined a set of machine learning (ML) and deep learning (DL) models to detect malicious websites using a dataset comprising 66,506 records of URLs. We engineered three different types of features including lexical-based, network-based and content-based features. To extract the most discriminative features in the dataset, we applied several features selection algorithms, namely, correlation analysis, Analysis of Variance (ANOVA), and chi-square. Finally, we conducted a comparative performance evaluation for several ML and DL models considering set of criteria commonly used to evaluate such models. Results depicted that Naïve Bayes (NB) was the best model for detecting malicious URLs using the applied data with an accuracy of 96%. This research has made contribution to the field by conducting significant features engineering and analysis to identify the best features for malicious URLs predictions, compare different models and achieve a high accuracy using a large new URL dataset.

## 1. Introduction

The creation of the World Wide Web (WWW) was revolutionary in the history of the Internet as it enabled anyone to access the Internet in a way that was previously not possible. Social networking sites and blogs made it easier for people to access, share, and communicate information, expertise, thoughts, and ideas. Businesses expanded and went global as companies extended their reach through the Internet. The Hypertext and Hypertext Markup Language (HTML) enabled the efficient creation of web pages, and the Uniform Resource Allocator (URL) became the mechanism used by browsers to locate, identify, and retrieve any published web resource. The number of web domains exploded with the increasing number of Internet users. This expansion of the web attracted cybercriminals who distribute malware and compromise the confidentiality, integrity, and availability of resources on the web. The number of malicious websites has increased dramatically in the past few years, with these websites often being created by cyber attackers to access innocent victims' devices in an unauthorized manner and even convert numerous computers into bots to launch cyberattacks on targeted organizations. Through techniques such as creating web domains that look like reputable business web domains, they fool people into surrendering their data, infect them with malware, and persuade them to purchase fake goods. Additionally, the number of fraudulent sites is rising, a case in point being COVID-related sites giving false information to mislead people. Thus, with the rise of malicious URLs, researchers have presented defense approaches that are blacklisting-based, anomaly-based, and heuristic-based. Blacklisting-based methods rely on the construction of a blacklist of malicious URLs and using it to identify them. However, this method is only effective against known malicious URLs and could easily be circumvented as it requires exact matching [1]. Anomaly-based methods build classification models using discriminative rules or features to identify harmful URLs. The challenge posed by these methods is selecting the right discriminative features for it to be effective [1]. Heuristic approaches create signatures of known attack patterns to scan websites. The major downfall of these approaches is that they are unable to detect new attacks and cybercriminals can easily evade the predefined signatures [2].

Intelligent approaches, such as Machine Learning (ML) and Deep Learning (DL), have recently become widely popular in the research community to tackle challenges in various domains [3]. Such intelligent approaches are increasingly applied to data in order to predict the future and gain key insights that aid the decision-making process. ML and DL are used in a plethora of applications mainly for the prediction of new data output from historical data inputs. ML is a field in Artificial Intelligence (AI) that uses data and algorithms to imitate the way humans learn. It improves over time and with experience. DL, a subfield of ML, focuses on how the human brain works and simulates the structure of the brain and connecting neurons. Statistically, these intelligent algorithms can be used in classification problems, where the predictive models conjecture the class label of a given input, as well as in regression problems that entail predicting a continuous output value based on provided inputs. Network security is a very challenging field that increasingly utilizes the power of ML and DL to protect the confidentiality, integrity, and availability of networks and data. In this paper, we focus on using these intelligent techniques to detect malicious URLs. These techniques depend on the type of data and the set of extracted features that they are trained on. Thus, it is necessary to extract the right set of features to discern malicious and benign URLs using features engineering and selection methods.

Our aim is to create a model with high accuracy that prevents the attackers from conducting fraudulent activities and deceiving others by creating deceptive and misleading websites and domains. This paper focuses on using a new dataset, extracting useful features from it, and selecting the most optimal features using different features selection techniques to build and compare the accuracy of a set of intelligent models. Hence, the focal point of our study was to extract the most essential features for malicious URL classification and to identify which features are the most effective in carrying out accurate predictions on identifying malicious URLs.

The paper makes the following key contributions:

- Extracted a total of 39 features belonging to lexical-based, content-based, and network-based categories. These three categories of features were engineered from a recently published dataset, in a way to the best of our knowledge have not been engineered in previous studies.
- Compared with other studies, we reached comparative performance results with larger size of URL samples and a reasonable number of features that were selected using different features selection algorithms.
- Conducted a comparative performance evaluation of different ML and DL models for classifying URLs as benign or malicious. Random Forest (RF) and Naïve Bayes (NB) where applied as ML models, while Convolutional Neural Network (CNN) and Long Short-Term Memory (LSTM) were applied as DL models. The best performance was achieved using the NB classifier (with an accuracy of 96%).

The paper is organized as follows: Section 2 provides a review of the literature and highlights our contributions.  Section 3 discusses the methodology used in our experimentation, including the dataset description, the preprocessing stage, the intelligent methods applied, and the evaluation metrics used.  Section 4 discusses and compares the experimental results of the work. Finally, Section 5 concludes the paper and examines the possible future work.

## 2. Literature Review

Several studies have been performed with the aim of detecting malicious URLs using data mining approaches, some of which are reviewed and summarized below. There are various types of features that can be extracted from the URL. The most used features can be classified into three main classes: Lexical-based, Content-based, and Network-based. Lexical features are collected through lexical scanning and are obtained from the URL name/URL string itself. Content-based features reflect the web page content, including its HTML tags, iframes, lines, hyperlinks, JavaScript functions, and keywords that are mostly extracted by crawling the web page using tools such as Selenium WebDriver. Network-based features combine Network, Domain Name systems (DNS), and host-based features and include latency, DNS query data, domain registry data, payload size, WHOIS queries information about domain names, and IPs. The following is a summary of research papers grouped according to the class of features used in the studies where we considered three classes of features which are lexical, content-based, and network-based features.

Urcuqui et al. [5] focused on the recognizing malicious URLs using ML techniques. Their goal was to compare the performance of the classifiers when choosing specific features selected through CfsSubsetEval (CSE) and the performance when all the features were used. The dataset used was obtained from [6] and contained 1,781 records and 20 URL lexical, content-based, and network-based features. The features selection process was conducted using the CSE method, which entails scaling the importance of attributes based on the predictive capability of attributes as well as their redundancy degree. The authors used four classifiers, namely, K Nearest-Neighbour (KNN), RF, BayesNet, and J48. The performance measures were precision, recall, and accuracy. The best result achieved was by RF with 95% accuracy, 95.4% recall, and 95.3% precision. Along the same line, Kumi et al. [7] proposed a method to classify URLs as either malicious or benign using a dataset collected by Manjeri et al. [6]. The dataset was divided into 1,565 Benign URLs and 216 Malicious URLs. The researchers applied oversampling on the data to achieve the same number of samples of both the classes using the Synthetic Minority Over-Sampling (SMOTE) technique and a features selection technique named Recursive Feature Elimination (RFE). They used five classification algorithms, namely, RF, Decision Trees (DT), Logistic Regression (LR), KNN, and Support Vector Machine (SVM), and the evaluation metrics used were accuracy, precision, and recall. The highest accuracy of 96% was produced by RF.

Some studies did not use all three types of features (lexical, content, and network). For instance, McGahagan et al.'s [8] research used only lexical and content features to categorize URLs as malicious or benign using a data mining approach known as classification based on association (CBA). The researcher's dataset was collected from several sources. Benign URLs were collected from the top 500 sites by crawling from ALexa top sites. Malicious URLs were collected from VxVault, OpenPhish, and URLhaus. CBA consists of a classifier builder (CBA-CB) and rule generator (CBA-RG). They used correlation as a features selection technique, with the number of features selected being 12. They used precision, confusion matrix, recall, and accuracy to evaluate the proposed approach. The best result was a 95.83% accuracy rate achieved by the CBA model with low false positive and negative rates in the classification of URLs. Patgiri et al. [9] aimed to build several detection models using unsupervised and supervised learning algorithms over several sampling and feature transformation scenarios. They based their research on lexical and content-based features, removing any additional features by using the eXtreme Gradient Boosting (XGBoost or XGB) algorithm and Gini Impurity to calculate feature importance resulted in the selection of 34 features. They created three scenarios for sampling for the imbalanced dataset, namely, No-sampling, Under-sampling, and Over-sampling. The researchers built two models based on unsupervised learning algorithms, which are Autoencoders (AE) and SVM. They also built eight models based on supervised learning algorithms, which are Adaptive Boosting (AB), Extra Trees (ET), RF, Gradient Boosting (GB), XGB, Bagging Classifier (BC), the Voting classifier (V), and KNN. They achieved an accuracy of 98.03% by the V classifier. Kim et al. [10] used lexical and network-based features and applied ML to detect malicious URLs. The team collected a dataset of malicious and nonmalicious URLs considering only the lexical and network-based features. To extract these features, the team first used tokenization to break the URL into tokens or words followed by the features extraction method to calculate the length of the string. A bag of words was applied to obtain the number of sensitive words. RF and SVM were built using three types of train/test split: 80/20, 70/30, and 60/40. The 80/20 split gave good results with RF outperforming SVM, obtaining an accuracy of 93.3%.

In a study that focused only on extracting malicious paths based on content-based features Khan et al. [11] proposed a framework called WebMon. It consists of a database, queue server, and containers with a WebKit2 browser engine. The researchers introduced 11 content-based type features to detect exploit kits (EKs) and malicious URLs. EKs descript malicious codes to get system privileges on the victim's PC and install malware. The dataset contained 160 records, with 55 malicious records and 105 nonmalicious records. The detection accuracy of WebMon was evaluated using YARA rules and the ML classifier. YARA rules detect unique properties while ML comprehensively detect previously hidden exploit kit (EKs) variants that avoid pattern detection. For example, obfuscated pages are mainly exposed by an ML classifier and nonobfuscated pages are found by the YARA rules. The used ML classifiers were RF, SVM, NB, LR, Bayes Net, and J48. The proposed model (WebMon) has a detection accuracy rate of 98%.

Some researchers used only lexical features in their studies to assess intelligent models. Atrees et al. [12] used the chi-square and ANOVA F-value to select the most important lexical features from various datasets. The researchers' goal was to discover the most significant features of URLs. The researchers extracted 106 lexical features and used two scoring functions for features selection on each dataset to find the best 60 features. They found 47 features that were common to the two datasets. The researchers used several ML algorithms, including LR, KNN, SVM, and Ensemble learning algorithms. They measured performance using the confusion matrix, accuracy, precision, recall, and F1 score. The weighted average accuracy obtained for the model was 96.60%. To enhance the detection rate of malicious URLs, authors in [14] introduced a combination of ML approaches with AdaBoost as an ensemble learner algorithm. With the usage of phishing URLs dataset from UNB repository, results showed an improvement in the detection rate where decision tree (J48) algorithm with AdaBoost achieved the highest accuracy of 97.89%. In another study conducted by Wei et al. [15], the researchers developed a method to classify the URLs according to whether they were normal or malicious using the Neural Network. The dataset used in this research was CICANDMAL2017 [16], and 8 lexical features were mined from URL. The researchers used a feed-forward neural network with multiple hidden layers to detect the type of URL. Neural network algorithms were able to classify the URL attack type according to one of five types of malicious URLs, namely, spam, phishing, defacement, malware, and benign. After several runs with different dataset sizes and different numbers of hidden layers, the best results were obtained with 500 data rows and 25 hidden nodes; the neural network was able to detect achieving an accuracy of 98.48%.

Along the same lines, Saleem Rajaet al. [17] proposed a model to detect malicious URL addresses using CNN. The level of accuracy achieved was 99.98% using the CNN model. Chen et al. in [18] proposed a model for detecting malicious URLs based on CNN, with the usage of autoencoder to represent URLs. The features have been represented by using natural language process (NLP) for URL tokenization and then training an autoencoder for representing URL features. The experimental results showed high performance of 98.2% in terms of the accuracy on HTTP CSIC2010 dataset. Furthermore, Nargesian et al. [19] carried out a study to detect malicious URLs using lexical features. The dataset used was the UNB dataset. The number of features selected was 27 and were selected by correlation analysis, these were reduced to 20. The classifiers used in this study were RF, LR, kNN, Naïve Bayes (NB), and Support Vector Classification (SVC). The results showed that the RF classifier outperforms other classifiers in terms of accuracy by 99%. Li et al. [20] sought to identify malicious URLs from benign URLs using ML. The dataset contained a total of 26,054 URLs, half of which were malicious. It contained 41 features that were a mix of Alexa-based, network-based, and content-based features. Alexa provides services to Amazon. It organizes the behaviors of users on the Internet using big data and monitors the traffic of all domains. The researchers used two features selection techniques: the ANOVA and XGBoost algorithms. This study used four classic ML algorithms—SVM, KNN, XGBoost, and DT. As evaluation metrics, it used precision, recall, F1_score, and accuracy. The dataset that used the XGBoost model achieved 99.98% accuracy using the first 9 features only.

Features engineering is a fundamental concept in data science, and it still remains a time-consuming and challenging process [21]. In URL classification problems, most studies focused on improving ML and DL classifiers. Few studies focused on features selection and extraction [8, 22–27, 37]. Most of these studies either applied lexical features only, content-based only, or network-based. They did not apply combination of all these three types in order to perform predictions using ML/DL models. Taking into account the idea that features engineering plays a vital role in the performance of classifiers, our paper aims to extract different URL features, namely, lexical, network-based, and content-based features to help the classifier identify malicious URLs accurately. We apply these methods on a dataset constructed by Alkhudair et al. [4], which to the best of our knowledge has not been used in the papers mentioned above to apply ML and DL classification models. Furthermore, we carry out a series of experiments to select the features selection technique that yields the best results with a high level of accuracy. Based on the set of experiments performed, we select a combination of three features selection techniques, namely, ANOVA, chi-square, and correlation, to build the final dataset to feed to the intelligent models. In our research, we apply several ML and DL classifiers and provide comparative performance analysis. Combining more than one category of features yields better results.

Some researchers have investigated other features in order to analyze and enhance the models' performance. Mamun et al. [46] used click traffic data collected from Bitly over 600,000 short URLs and analyzed the traffic of the malicious and nonmalicious URLs, the best achieved result was using Random Tree algorithm with an accuracy of 90.81% and F-measure value of 91.3%. Al-Aidaroos et al. [47] focused on forwarding-based features of the URLs in the social media networks. Totally, 100,000 messages from Sina Weibo platform were collected and used to build 3 ML models: Bayes net, J48, and RF. The average achieved accuracy was 83.21%, the average F-positive rate was 10.03%, and the average F-measure was 86%. Similarly, Dogra et al. [48] also considered the pattern of the URLs posting in twitter social media platforms by analyzing the behavior of the URLs posting users and URLs clicking users. Using twitter APIs combined with Bitly APIs they collected around 7 million tweets that contain shortened URLs created by Bitly and tried different sets of features including Average clicks, Posting count, Median followers, Median friends, Score function Score Category. The best achived accuracy reached 86%.

Comparison with previous studies is challenging because different datasets, features, classifiers, and performance metrics were used. According to the literature review, the best results were achieved by Li et al. [20] with an accuracy rate of 99.99% using CNN. However, their dataset contained 26,054 records, which is less than half the size of our dataset that contained 66,506 records and still achieved a compatible result with an accuracy level of 96.01%. Further, they did not use lexical features of the URLs to make predictions and only investigated the network-based, and content-based features. In our paper, we focused on investigating combination of network-based, lexical-based, and content-based features, which are essential in order to effectively make URL predictions [9].

**Figure 1:**
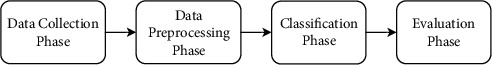
Generic flow diagram for methodology.

We conclude from the previous studies that a general methodology was applied for building the intelligent models as depicted Figure 1.

## 3. Methodology

**Figure 2:**
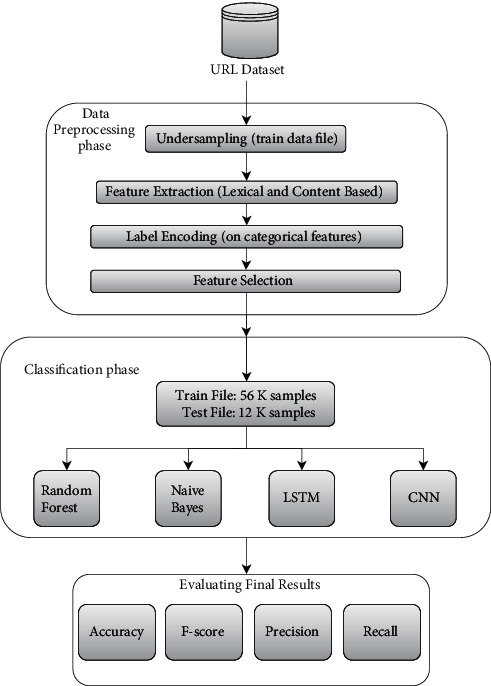
Experiment methodology.

Our research examined and classified websites as benign or malicious using a new dataset and extracting useful features from it to obtain a high level of accuracy. We followed a consistent methodology that is included in all studies, which includes 4 main set-ups: Data acquisition, Preprocessing, Classification, and Evaluation. First, we analyze the dataset to understand the existing features and determine the required features. Second, we extracted more useful features from the dataset. We then applied further preprocessing techniques to prepare the data to build both ML and DL models. Finally, we analyzed the results.  Figure 2 shows a diagrammatic representation of the methodology carried out in experimentation and discussed in more details in the following subsections.

### 3.1. Dataset Description

The dataset employed was the Malicious and Benign Webpages Dataset, a publicly available dataset released by Singh and Kumar in 2020 [4]. The data were collected by crawling the Internet using the Mal Crawler tool [28], and the labels were verified (as good or bad) using Google Safe Browsing (API). The dataset contained various attributes from websites, namely, URL, IP address, JavaScript code, obfuscated code, geographical location, top-level domain, and HTTPS, all of which can be useful for classifying a web page as either malicious or benign. The dataset also included raw page content including the JavaScript code that can be used to extract further attributes. The dataset contained two sets: one set of webpages represents the train data which comprised 1.2 million records and 11 attributes and the other set of webpages represent the test data, which comprised 0.364 million records and 11 attributes. In the train dataset, 27,253 were labelled malicious and 1,172,747 were labelled benign. In the test dataset, 353,872 URLs were labelled benign and 8,062 were labelled malicious.  Table 1 shows a short overview of all the features involved in this dataset.

### 3.2. Preprocessing Phase

**Figure 3:**
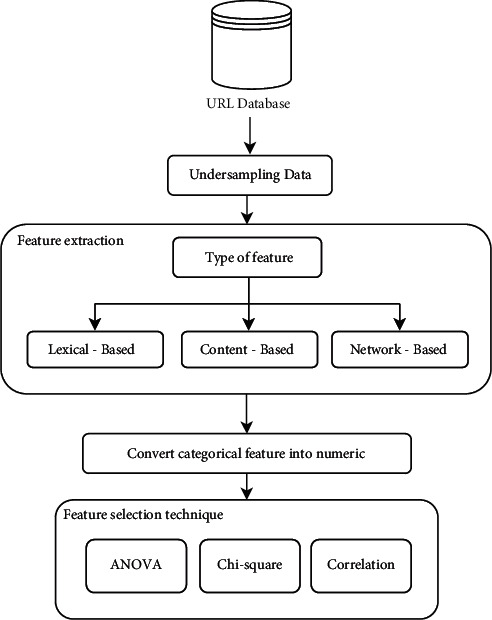
Diagrammatic representation of preprocessing phase.

A fundamental phase in ML and DL models is the preprocessing phase, which prepare the data for the application of classification techniques.  Figure 3 demonstrates the preparation steps including undersampling, features' extraction, label encoding, and features' selection. These steps are explained in more detail in the following sections.

#### 3.2.1. Undersampling the Train Dataset

Randomized undersampling is a technique used to solve a class imbalance problem in the dataset. It refers to the process of decreasing the number of samples in the majority class to balance it out with the minority class in the dataset. In this work, our train dataset contained imbalanced classes as most of the sample records were benign (1,172,747 out of 1.2 million), and only 27,253 were labelled malicious. Following the randomized undersampling process, the train dataset ended up comprising 27,253 malicious records and 27,253 benign records; thereby, bringing the total number of records in the train dataset to 54,506. In the case of the test data file, from the 300K records present in the original file, 12000 records were selected at random. Hence, all in all, the train data file contained 54,506 records, and the test data file contained 12000 records.

#### 3.2.2. Features' Extraction

After analyzing the dataset, we applied different techniques to engineer and extract the most useful features from the dataset. Apart from the network-based features that were already present, we extracted URL-based lexical features and web page content-based features.

For extracting the lexical features that reflect the textual properties of URL, namely, the special characters, path extension, path, host, and URL lengths, etc., we used several libraries in Python and Scikit Learn to extract the following features:

- Special Characters: generally, for URL encoded attacks, malicious users utilize special symbols to bypass validation logic. Hence, we counted the presence of each of these symbols, e.g., the dot, @, &, /, −, = , \, and ? in the URL.
- Special words: we checked the presence of special names in the URL such as ebayisapi, getImage, log, and jpg and saved it as a Boolean feature.
- Path length: the path represents the exact location of a file, page, post, or other assets. For example, http://www.astonmartin.co.uk/models/index.html, http://www.astonmartin.co.uk comprises the hostname portion of the URL and models/index.html represents the path. Also, we extracted the length of the path, number of dots in the path, and slashes.
- Host length and digits in host: the host refers to the name of the webserver. host_Presence_of_digit is a new feature that checks the presence or absence of digits in the host as a Boolean. Using python, we first extracted the hostname from the URL feature, and we then extracted the host length and digits in the hostname from this extracted feature.
- URL length: represents the total length for the whole URL.
- Digit and letter count: count_digit and count_letter are new features that represent the total number of letters and digits in the URL.

For the content-based features that mainly refer to the JavaScript functions and HTML tags that could be utilized by attackers to exploit websites, we extracted the following features:

- Presence of iFrame on the website: the HTML tag called iFrame has been used in a number of attacks to download malicious JavaScript exploits [23, 29]. In our chosen dataset, we searched for the presence of this tag in the content features and stored its presence or absence in a new feature called presence_iFrame as a Boolean.
- Presence of suspicious JavaScript Function:
- Eval function: this function is used by attackers to generate malicious code in malicious websites at runtime to thwart detection [30]. In the dataset, we searched for the presence of this function eval() in JavaScript code in the content features and stored its count in a new feature called count_eval as a numerical attribute.
- Escape function: hackers generally encode malicious code using this function to later decode it and initiate attacks. In the dataset, we searched for the presence of this function escape() in JavaScript code in the content features and stored its count in a new feature called count_escape as a numerical attribute.
- Unescape function: after encoding the malicious code using escape(), attackers generally decode it using the unescape() function and eventually initiate attacks. Therefore, the number of escape and unescape functions is a good indicator of malicious activity [31]. In the dataset, we searched for the presence of this function unescape() in JavaScript code in the content features and stored its count in a new feature called count_unescape as a numerical attribute.
- Find function: in order to decrypt malicious code at runtime, attackers integrate find() along with eval() and unescape() functions [31]. Therefore, we searched for the presence of this function find() in JavaScript code in the content features and stored its count in a new feature called count_find as a numerical attribute.
- Exec, Search, and Link functions: we searched for the presence of these further suspicious functions used by attackers in JavaScript code in the content features and stored its count in new features as numerical attributes.
- Total count of the suspicious functions: since all the above-mentioned functions are used by attackers for cross-site scripting and malware distribution [12], we added up the counts of all these functions that were present in the JavaScript code in the content features and stored its final count in a new feature called count_all_functions as a numerical attribute.
- Presence of Windows.open function: the Windows.open functions are generally used for ads popups and can be used to inject attacks on malicious sites. We checked the presence of this popup in the content and stored it as a Boolean value.
- Line count: the total count of the lines presents in the content. It was extracted by checking the presence of the newline symbol “\n” in JavaScript code in the content features and stored its count in a new feature called lines_count as a numerical attribute.

The host-based features, namely, the IP address, geographical location, who_is properties, etc. are already present in the dataset. At the end of the features extraction process, the features presented in Table 2 below were extracted from the dataset.

#### 3.2.3. Converting Categorical Features to Numerical

After the undersampling of data and extraction of useful features, we performed further preprocessing namely label encoding to convert all the categorical data into a numerical form and convert all categories into numbers. The target variable (named label) was categorized as good or bad. After the preprocessing where good was made as 0 and bad as 1, the variable was converted to numeric form. Similar steps were applied for who_is and HTTPS features.

#### 3.2.4. Features' Selection

Features selection is the technique used to select a subset of the most relevant columns or features in a dataset. Features selection helps the ML/DL algorithms be more effective and efficient as it uses less space and reduces time complexity. It also reduces the high data dimensionality. Also, irrelevant features can mislead ML/DL algorithms and result in poor performance. For our experimentation, we considered three features selection techniques:

*(1) Analysis of Variance (ANOVA)*. is a popular statistical method for features selection. ANOVA is used to analyze the variation in a response variable that is measured under various conditions specified by discrete factors (classification variables) [32]. The test statistic for ANOVA is given by (1) below:

(1) $$\begin{array}{c} F=\frac{\sum_{n_{j}}{\left({\bar{X}}_{j}-\bar{X}\right)}^{2}/\left(k-1\right)}{\sum \sum {\left(X-{\bar{X}}_{j}\right)}^{2}/\left(N-k\right)}. \end{array}$$

*(2) Chi-square*. is a statistical procedure used by researchers to measure the difference between the observed results and the expected outcome. In other words, it is used to check the independence of two variables [33]. Where O signifies the observed value and E reflects the expected value, and the chi-square measures how these two variables deviate from each other using :

(2) $$\begin{array}{c} \chi^{2}=\sum \frac{{\left(O_{i}-E_{i}\right)}^{2}}{E_{i}}. \end{array}$$

This formula gives a chi-square value that can be used to decide if the feature is dependent on the response and if it can be selected. The higher the chi-square value, the more dependent the feature, which means that it can be selected for application in the model.

*(3) Correlation*. is a statistical term that is used to measure how close two variables are to having a linear relationship with each other. It is used to find the association between all the features and the target class feature. If the features are linearly independent (uncorrelated), then the correlation coefficient is 0. If the features are linearly dependent (correlated), then the correlation coefficient is ±1. Assuming that there are two samples *X* and *Y* where sample *X* contains *m* sample observations (*x*1, *x*2, .…, *xm*) and sample *Y* contains *m* sample observations (*y*1, *y*2,…, *ym*) [34], the Pearson correlation coefficient is given by Eq. (3) below:

(3) $$\begin{array}{c} r=\;\frac{\left(N\sum x_{i}y_{i}\;-\;\sum x_{i}\sum y_{i}\right)}{\sqrt{Nx_{i}^{2}-{\left(\sum x_{i}\right)}^{2}}\sqrt{Ny_{i}^{2}-{\left(\sum y_{i}\right)}^{2}}}. \end{array}$$

**Figure 4:**
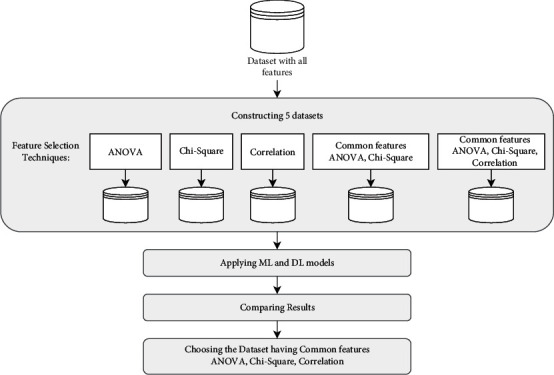
Selecting features.

In our work, 39 original features of the dataset and 1 classification label feature were used. To select the most effective method for features selection, we performed a set of experiments by constructing 5 instances of the dataset using a subset of features, as depicted in Figure 4. The first three instances of the datasets were constructed using correlation, ANOVA, and chi-square respectively to score the best features for features selection using these individual methods. The ranked lists of features were generated based on these scores to select a subset containing the best discriminative features. The fourth instance of the dataset was constructed by taking the common best features from the dataset instances that used ANOVA and chi-square as scoring functions for features selection. The last instance of the dataset was constructed by taking the common best features between the three constructed dataset instances that used correlation, ANOVA, and the chi-square. We then analyzed and compared the results of all the five instances of the dataset after applying DL and ML models, namely, CNN, LSTM, NB, RF, on each dataset. After analyzing the result, we selected the dataset containing the common best features from the three methods: ANOVA, chi-square, and correlation.

Table 3 shows the final set of selected features.

### 3.3. Classification Phase

In order to train and test the dataset for malicious URL detection, we evaluated several ML and DL models such as NB, RF, LSTM, and CNN [35].

(i) RF is one of the supervised ML techniques, and it is a collection of random trees. RF is a decision tree algorithm that extends the popular decision tree technique by combining a greater number of decision trees. The goal of this method is to reduce the variance of the innovative decision tree. RF is one of the most popular classifiers due to its ease of use and adaptability since it can handle both classification and regression problems [38].
(ii) NB is a probabilistic model that is based on the Bayesian theorem. The influence of a feature on a class is assumed to be independent of the values of other features by naive Bayes classifiers. This conditional independence streamlines the algorithm while maintaining accuracy. The NB equation is presented by equation (4) below [38].
  (4) $$\begin{array}{c} P\left(y|x\right)=\frac{p\left(x|y\right)p\left(y\right)}{p\left(x\right)}. \end{array}$$
(iii) CNN is a type of DL technique that has shown to be highly useful in recent years due to its ability to exchange weights by exploiting the local connections between surrounding values in both image and sequence data. 2D or 3D convolutional layers are typically utilized with the pictures. However, a 1D convolutional layer has been employed to work with text, which has shown to be highly successful, mainly when dealing with time-series or sequence data [39, 40]. CNN reduces the requirement for manual features extraction because the network learns the features immediately. CNN can be retrained to do new tasks that build on previously trained networks [41].
(iv) LSTM is a type of recurrent neural networks (RNN) that can learn long-term dependencies. The goal of designing this algorithm is to avoid the problem of long-term dependency [42]. Although LSTMs have a chain-like structure, the repeating module has a different structure [43].

### 3.4. Evaluation Criteria

A number of metrics can be used to compare the performance of different ML and DL classifiers. We used confusion matrix, accuracy, precision, recall, and F-score to evaluate and compare the performance of the applied models. The confusion matrix, which is used to calculate the accuracy of the classifier, is a four-way table of predicted and actual classifications done by the models.

- True Positive (TP): correctly predicting benign URLs as benign.
- False Positive (FP): incorrectly predicting benign URLs as malicious.
- True Negative (TN): correctly predicting malicious URLs as malicious.
- False Negative (FN): incorrectly predicting malicious URLs as benign.

Accuracy is the ratio of correctly predicted classes to the total number of instances and is computed by (5) below:

(5) $$\begin{array}{c} \text{Accuracy}=\frac{TP+TN}{\left(TP+TN+FP+FN\right)}. \end{array}$$

Precision is finding out how many of the classes predicted as positive are actually positive and is computed by (6) below:

(6) $$\begin{array}{c} \text{Precision}=\frac{\mathrm{TP}}{\left(\mathrm{TP}+\mathrm{FP}\right)}. \end{array}$$

Recall is the ratio of classes predicted correctly out of all the positive classes and is computed by (7) below:

(7) $$\begin{array}{c} \text{Recall}=\frac{\mathrm{TP}}{\left(\mathrm{TP}+\mathrm{FN}\right)}. \end{array}$$

F-score helps measuring both recall and precision simultaneously and is computed by (8) below:

(8) $$\begin{array}{c} \text{F}-\text{score}=\frac{2*\mathrm{Recall}*\mathrm{Precision}}{\mathrm{Recall}+\mathrm{Precision}}. \end{array}$$

## 4. Results and Discussion

### 4.1. Experimental Setup

As discussed in the methodology section, the dataset underwent a preprocessing phase, where different steps, namely, features' extraction, label encoding, features' selection, and then undersampling the train data were implemented. Finally, the dataset contained 15 features and 1 target label, 54,506 samples of URLs for the training set, and 12000 samples of URLs for the testing set labelled either benign or malicious. At the end of these steps, the dataset was ready to feed into the ML models, namely, the RF, NB, as well as different DL models, namely, the CNN and LSTM. All the models were built using python and Scikit Learn. The experiments for the DL models were carried out in a Google Research product called the Google Colab environment. Whereas, the ML models were built in Jupyter Notebook python coding platform. Our DL models are trained for 1,000 epochs, wherein each epoch all the features, along with their corresponding target label (0 or 1), are fed in batches of 32. We used a function called early stopping that stops the iteration before 1,000 epochs since too many epochs lead to overfitting and too few may lead to underfitting. Hence, early stopping ensures that the model stops training once its performance stops improving on the training set provided. The early stopping function monitors the model's loss and terminates iteration when the loss does not decrease.  Table 4 below demonstrate the parameters settings applied for all models. The parameters were defined according to a pilot study that showed the best performance using such parameters.

## 5. Results

In order to demonstrate the effectiveness after training, we validated our models on a test set that contained 12,000 URLs. We evaluated our models using various evaluation metrics, namely, accuracy, precision, recall, and F1 score.  Table 5 shows the classification results obtained for all the models.

**Figure 5:**
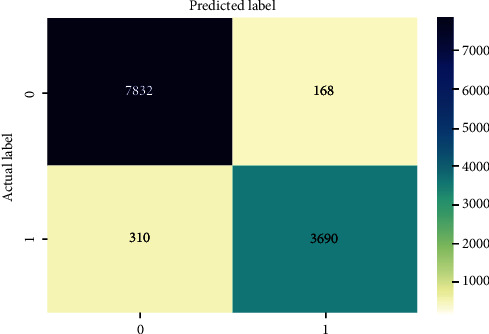
Performance analysis of NB.

From the results table, we see that all the models gave very close values. Looking at Figure 5, we see that NB achieved slightly higher values than the other models, achieving accuracy of 96.01%. The highest achieved precision of 95.64% was by NB, which was followed by RF, CNN, and then LSTM, which obtained precision between 87.24% and 87.31%. However, the recall achieved by NB was the lowest as all the other models achieved a recall of 100%. Finally, the F-score achieved by all the models was in the similar range from 93.18% to 93.93%.

**Figure 6:**
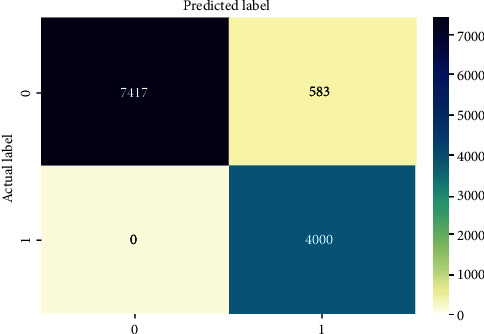
Performance analysis of RF.

**Figure 7:**
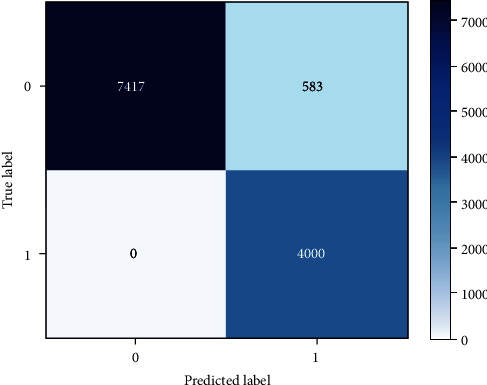
Performance analysis of LSTM.

**Figure 8:**
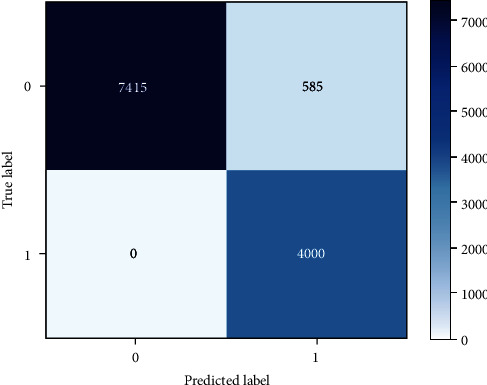
Performance analysis of CNN.

The correct identification of benign URLs is considered TP, whereas the recognition of malicious is identified as TN. Similarly, the wrong identification of malicious URLs as benign is referred to as an FN, and the wrong identification of benign URLs as malicious is called an FP. All of our models were trained independently, and then were assessed based on their confusion matrices. A model will be more accurate if it has more TP and TN (or fewer FN and FP) [45]. The confusion matrices associated with the models are presented in Figures 5–8. These confusion matrices are also used to calculate metrics such as precision, recall, and the F1 score. From these matrices, it can be seen that NB performed better than other models. In NB, the instances of TP and TN were more, compared with the other models.

As seen from Table 5, NB obtained the highest accuracy and precision but the lowest recall. NB uses the concept of conditional probability formulated by Bayes Theorem. In more concrete terms, each attribute/feature is considered independently on the class. Therefore, NB is known to work well in classification problems largely because it reduces the curse of dimensionality. The conditional probability ensures that the outcome of one feature on the class label does not interfere with the outcome of another attribute on the class label. Likewise, in our experiment, NB was able to correctly predict 7832 benign labels, as well as correctly predict 3690 malicious labels in the test dataset. However, it incorrectly predicted 310 malicious websites as benign and 168 benign websites as malicious as shown in Figure 5. The per class values of accuracy, precision, and recall are included in Table 6.

The second highest accuracy and recall were obtained by RF, but it obtained a low precision score. RF is an ensemble model that contains multiple decision trees that are known to split the data into data groups based on the features until a prediction is reached. RF improves the model performance because it decorrelated the features by splitting the tree into feature subsets at each consecutive node. This ensures that the variance is averaged out and the performance obtained in predicting the classes is high. Hence, our RF model was able to correctly predict 7417 benign URLs as well as 4000 malicious URLs. On the other hand, it incorrectly predicted 583 benign URLs as malicious and did not incorrectly predict any malicious URLs as benign as seen in Figure 6. Therefore, RF can be a good choice to make malicious URLs predictions as it was successfully able to identify all malicious domains correctly.  Table 7 gives the per class metrics for RF.

Following that, LSTM came behind and achieve similar results. LSTMs are a more sophisticated version of RNNs, which have the advantage of remembering past data more easily. The use of LSTM is frequently preferred over RNN in practice since it is more computationally efficient where it was invented to solve a problem that standard RNNs have, namely the vanishing gradient problem. However, in our experiments, the outcomes of these two models were identical, indicating that there is no difference between the models in our case unless we are dealing with a time-series situation with short/long-term dependency. The results of LSTM showed a correct prediction of 7417 benign labels, as well as correctly predict 4000 malicious labels in the test dataset. However, it incorrectly predicted 583 benign websites as malicious and did not incorrectly predict any malicious URLs as benign as shown in Figure 7. Further, the different metrics per class are included in Table 8 for LSTM.

The lowest accuracy was obtained by the CNN model, which is a type of neural network with one or more convolutional layers that are typically used for image processing, classification, and segmentation. To process and train the neural network, CNN needs a massive dataset that could be one factor for being the least performance model compared with other models in this study. CNN is also more typically associated with visual imagery than with tabular data, which could be another factor. In our experiment, CNN was able to correctly predict 7415 benign labels, as well as correctly predict 4000 malicious labels in the test dataset. However, it incorrectly predicted 585 benign websites as malicious and did not incorrectly predict any malicious URLs as benign as shown in Figure 8.  Table 9 gives the per class metrics for CNN.

## 6. Conclusion and Future Work

With the growing number of web domains, there has been a surge in the number of malicious URLs generally used by cybercriminals to inject malicious code into victims' devices, thereby compromising the confidentiality, integrity, and availability of systems. Therefore, there is an urgent need for detection methods to evolve and recognize the ever more sophisticated methods being used by attackers to target victims. Identifying ways to use intelligent methods to tackle this issue has become a significant research area. Many researchers have focused on building classification models. In this paper, we focused on features engineering and building different models, namely, RF, NB, CNN, and LSTM to analyze and compare to achieve a high level of classification accuracy. We evaluated the models' performance using a number of matrices including accuracy, precision, recall, and F-score. The final features used in the dataset for experimentation contained a mix of lexical, content-based, and host-based features which were selected by performing three features selection techniques, namely, ANOVA, chi-square, and correlation. The results of the experiments show that NB obtained the highest accuracy of 96.01%, followed by RF and LSTM with an accuracy of 95.1%. A plethora of features were extracted, and different sets of experiments were performed in this research. Future work can focus on extracting one content-based feature that sometimes improves accuracy: the top keywords feature of the website content. Some researchers have used the term frequency-inverse document frequency (TF-IDF) technique to extract the keywords. However, in our research, this technique produced numerous features, making it difficult to process and apply them in intelligent models due to memory constraints. Hence, in future work, we propose to extract the keywords feature from the dataset used and apply it in intelligent models to measure performance accuracy. Moreover, we suggest building real-time models capable of detecting new URLs and websites and classifying them in real time in order to stop suspicious websites before they get launched publicly [13, 36, 44].

## Figures and Tables

**Table 1:** Features present in the dataset.

#	Attribute	Description
1	url	URL of the website
2	ip_add	The IP address of the website
3	geo_loc	The geographical location where the website is being hosted
4	url_len	Length of the URL of the website
5	js_len	Length of JavaScript code present on the website
6	js_obf_len	Length of the obfuscated JavaScript code present on the website
7	tld	Top-Level domain of the website
8	who_is	WHO IS domain information is complete or no
9	https	Website is HTTPS protocol using
10	Content	Raw web page content with JavaScript code
11	Label	Label for indicating if the website is malicious or benign

**Table 2:** Final features in the dataset after features extraction.

#	Attribute	Description
1	url_len	Length of the URL of the website
2	geo_loc	The geographical location where the website is being hosted
3	tld	Top-Level domain of the website
4	who_is	Who is domain information is complete or no
5	https	Website is HTTPS protocol using
6	js_len	Length of JavaScript code present on the website
7	js_obf_len	Length of the obfuscated JavaScript code present on the website
8	count_link	Count appearance of JavaScript link() function in content
9	count_eval	Count appearance of JavaScript eval() function in content
10	count_exec	Count appearance of JavaScript exec() function in content
11	count_unescape	Count appearance of JavaScript unescape() function in content
12	count_search	Count appearance of JavaScript search() function in content
13	count_find	Count appearance of JavaScript find() function in content
14	count_escape	Count appearance of JavaScript escape() function in content
15	count_all_functions	Count of all the above 7 suspicious functions in content
16	Presence_iframe	The presence of the iFrame tag is checked in content
17	count_/	Count “/” symbols in URL
18	count_dot	Count “.” symbols in URL
19	Count_&	Count “&” symbols in URL
20	Count_@	Count “@” symbols in URL
21	Count_-	Count “−” symbols in URL
22	count_=	Count “=” symbols in URL
23	Count_?	Count “?” symbols in URL
24	Count_;	Count “;” symbols in URL
25	count_digit	Count total digits in URL
26	count_letter	Count total alphabetical letters in URL
27	presence_ebayisapi	Check presence in URL
28	presence_getImage	Check presence in URL
29	presence_jpg	Check presence in URL
30	presence_log	Check presence in URL
31	count_path_dots	Count dots in URL path
32	path_length	Length of the URL path
33	count_path_slash	Count backslashes in URL path
34	host_length	Length of the hostname in URL
35	host_Precense_of_digit	Check digits in the hostname
36	count_symbols	Count all symbols in the URL
37	presence_obfuscated_code	Check the presence of obfuscated JavaScript code
38	presence_Window.open()	The presence of Window.open() function is checked in content
39	lines_count	The number of lines of the content
40	Label	Label for indicating if the website is malicious or benign

**Table 3:** Final set of selected features.

#	Feature
1.	presence_obfuscated_code
2.	js_len
3.	js_obf_len
4.	count_All_Functions
5.	count_find
6.	count_unescape
7.	count_escape
8.	who_is
9.	https
10.	count_eval
11.	presence_iFrame
12.	count_search
13.	presence_Window.open()
14.	host_length
15.	Count_-

**Table 4:** Parameters' settings applied for all classifiers.

Model	Parameter	Value	
RF	Number of trees	100	

CNN	Activation function in hidden layers	ReLU	
	Number of neurons in output layer	1	
	Activation function in output layer	Sigmoid	
	Dropout	0.2, 0.5	
	Batch size	32	
	Number of layers	4	
	Number of neurons in hidden layers	32, 64, 64	

LSTM	Activation function in hidden layers	Tanh	
	Number of neurons in output layer	1	
	Activation function in output layer	Sigmoid	
	Dropout	0.1	
	Batch size	32	
	Number of layers	3	
	Number of neurons in hidden layers	8, 8	

**Table 5:** Results of the experiments.

	Accuracy	Precision	Recall	F-score
CNN	0.9513	0.8724	1.00	0.9319
LSTM	0.9514	0.8728	1.00	0.9321
NB	0.9601	0.9564	0.9225	0.9391
RF	0.9515	0.8731	1.0	0.9322

**Table 6:** Per class metric values for NB.

Class	Precision	Recall	f1 score
Benign	0.96	0.98	0.97
Malicious	0.96	0.92	0.94

**Table 7:** Per class metric values for RF.

Class	Precision	Recall	f1 score
Benign	1	0.93	0.97
Malicious	0.87	1	0.93

**Table 8:** Per class metric values for LSTM.

Class	Precision	Recall	f1 score
Benign	1	0.93	0.96
Malicious	0.87	1	0.93

**Table 9:** Per class metric values for CNN.

Class	Precision	Recall	f1 score
Benign	1	0.93	0.96
Malicious	0.87	1	0.93

## Data Availability

The data used to support the findings of this study are available from the corresponding author upon request.
